# Histone Deacetylase 6 Regulates Bladder Architecture and Host Susceptibility to Uropathogenic *Escherichia coli*

**DOI:** 10.3390/pathogens5010020

**Published:** 2016-02-14

**Authors:** Adam J. Lewis, Bijaya K. Dhakal, Ting Liu, Matthew A. Mulvey

**Affiliations:** 1 Department of Pathology, University of Utah, 15 N. Medical Drive E., Salt Lake City, UT 84112, USA; adam.lewis@path.utah.edu (A.J.L.); bijayadhakal@gmail.com (B.K.D.); 2 Huntsman Cancer Institute, University of Utah, 2000 Circle of Hope, Salt Lake City, UT 84112, USA; ting.liu@path.utah.edu

**Keywords:** histone deacetylase 6, bladder, neutrophil, invasion, UTI, urothelium, microtubules

## Abstract

Histone deacetylase 6 (HDAC6) is a non-canonical, mostly cytosolic histone deacetylase that has a variety of interacting partners and substrates. Previous work using cell-culture based assays coupled with pharmacological inhibitors and gene-silencing approaches indicated that HDAC6 promotes the actin- and microtubule-dependent invasion of host cells by uropathogenic *Escherichia coli* (UPEC). These facultative intracellular pathogens are the major cause of urinary tract infections. Here, we examined the involvement of HDAC6 in bladder colonization by UPEC using HDAC6 knockout mice. Though UPEC was unable to invade HDAC6^−/−^ cells in culture, the bacteria had an enhanced ability to colonize the bladders of mice that lacked HDAC6. This effect was transient, and by six hours post-inoculation bacterial titers in the HDAC6^−/−^ mice were reduced to levels seen in wild type control animals. Subsequent analyses revealed that the mutant mice had greater bladder volume capacity and fluid retention, along with much higher levels of acetylated α-tubulin. In addition, infiltrating neutrophils recovered from the HDAC6^−/−^ bladder harbored significantly more viable bacteria than their wild type counterparts. Cumulatively, these changes may negate any inhibitory effects that the lack of HDAC6 has on UPEC entry into individual host cells, and suggest roles for HDAC6 in other urological disorders such as urinary retention.

## 1. Introduction

Urinary tract infections (UTIs) are one of the most prevalent bacterial infections, with strains of uropathogenic *Escherichia* *coli* (UPEC) being the primary etiological agents [1,2]. The success of UPEC may in part be attributable to its ability to act as a facultative intracellular pathogen [3]. UPEC employ filamentous adhesive organelles, know as type 1 pili, to both bind to and invade the epithelial cells that comprise the bladder mucosa [4]. Within the cytosol of these host cells UPEC can multiply, forming large intracellular bacterial communities that can contain thousands of bacteria [5]. Alternatively, UPEC can enter a more quiescent state, bound by host vacuolar membranes and actin filaments [6,7]. These pockets of latent bacteria are protected from antibiotic treatments and many host defenses, and likely serve as important sources for the recurrent, or relapsing UTIs that afflict many individuals [8]. In both *in vivo* and cell culture-based assays, type 1 pili-mediated UPEC entry into host cells occurs by an actin- and microtubule-dependent zipper mechanism [5,9,10]. In addition to these cytoskeletal filaments, numerous other host factors have been implicated as regulators of the entry process. Among these is a multifaceted enzyme known as histone deacetylase 6 (HDAC6) [11].

HDAC6 is a non-canonical class IIb histone deacetylase that is localized primarily within the cytosol where it has multiple, non-histone binding partners and substrates [12]. Unlike most other HDAC enzymes, HDAC6 does not appear to have a substantial role in epigenetic regulation. In addition to nuclear export signals and a cytosolic anchoring region, HDAC6 contains a ubiquitin-binding zinc finger domain and two homologous catalytic domains. HDAC6 is known to interact with well over two-dozen proteins, with major substrates being the actin-binding protein cortactin, the chaperone Hsp90, and the microtubule subunit α-tubulin [12]. The use of pharmacological inhibitors, gene silencing approaches, and knockout mice indicates the involvement of HDAC6 in a wide range of biological processes. These include the modulation of inflammatory responses, the formation of focal adhesions, and the resolution of misfolded protein aggregates by the aggresome-autophagy pathway [12]. The dysregulation of HDAC6 is thought to contribute to a number of pathological conditions, including cancer, autoimmunity, and neurodegenerative diseases [12,13,14,15,16]. HDAC6 has also been implicated in the pathogenesis of infections caused by HIV, human T cell leukemia virus, Sendai virus, Influenza A, and other viruses [17,18,19,20,21,22,23]. Furthermore, in mouse models of septic shock, the deletion or inhibition of HDAC6 can prolong host survival [23,24,25].

In a previous study, we reported that pharmacological inhibition of HDAC6 activity or the silencing of HDAC6 expression greatly increased the amounts of acetylated α-tubulin within cultured bladder epithelial cells and significantly reduced UPEC invasion frequencies [11]. Overexpression of recombinant HDAC6 had similar inhibitory effects on UPEC entry, suggesting that an imbalance in HDAC6 activities could disrupt the invasion process. Accordingly, the inhibition or silencing of the HDAC6 activators Aurora A kinase and Casein Kinase II also impedes UPEC entry into host cells [11,26]. The acetylation of α-tubulin can stabilize microtubules and influence the recruitment and trafficking of microtubule-associated motors like kinesin [27,28,29]. Cumulative results from our *in vitro* assays suggest that HDAC6-mediated effects on the acetylation of α-tubulin alters the microtubule- and kinesin-dependent delivery of regulatory factors that modify actin dynamics at sites of UPEC entry [11]. Here, we set out to extend our *in vitro* findings using an *in vivo* UTI model system with mice that lack the gene encoding HDAC6. Our results indicate that the requirements for HDAC6 during the invasion process can be circumvented, but this conclusion is confounded by data showing that HDAC6 also affects the architecture of the bladder mucosa and smooth muscle layers, as well as the functionality of infiltrating neutrophils.

## 2. Results and Discussion

### 2.1. HDAC6^−/−^ Murine Embryonic Fibroblasts Are Resistant to Bacterial Invasion

**Figure 1:**
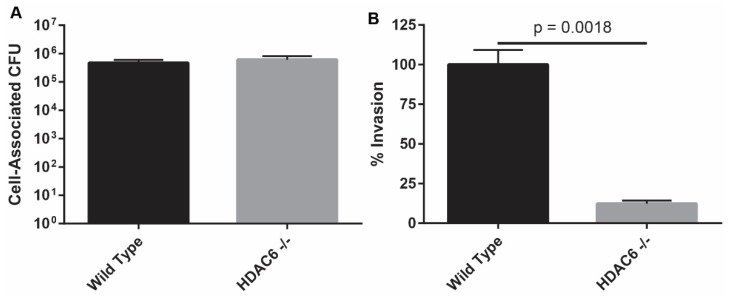
HDAC6 is critical for UPEC entry into cultured MEFs. (**A**) Graph shows total numbers of UTI89∆*hlyA* present in association with WT and HDAC6^−/−^ MEFs at 1-h post-inoculation of the cultures; (**B**) Levels of intracellular (gentamicin-protected) bacteria in HDAC6^−/−^ MEFs expressed relative to WT controls. Bars indicate mean values ± SEM from three experiments carried out in triplicate. *P* = 0.0018, versus WT controls, as determined by Student’s *t* tests with Welch’s correction.

Earlier research that implicated HDAC6 as a mediator of host cell invasion by UPEC employed pharmacological inhibitors and small interfering RNA with bladder cancer cell lines (5637 and T24 cells) or normal primary bladder epithelial cells [11]. To extend these findings with a system in which HDAC6 expression is completely ablated, we tested the ability of UPEC to invade murine embryonic fibroblasts (MEFs) that were derived from wild type (WT) or HDAC6^−/−^ mice. Published and ongoing work from our group and others indicates that UPEC and related pathogens can highjack host receptors and signaling pathways that are common to many host cell types, including urothelial cells and embryonic fibroblasts (e.g., [30,31,32,33,34,35]). Experiments described here used a derivative of the reference cystitis isolate UTI89 that lacks the pore-forming toxin α-hemolysin (HlyA). Deletion of *hlyA* does not interfere with the ability of UPEC to colonize host cells or the murine urinary tract, but does limit UPEC-induced damage to host cells and tissues [36]. UTI89∆*hlyA* bound WT and HDAC6^−/−^ MEFs similarly (Figure 1A). However, the ability of the bacteria to invade host cells lacking HDAC6 was dramatically compromised, as determined by gentamicin protection assays (Figure 1B). These results are in line with our previously published findings, confirming that HDAC6 can facilitate UPEC entry into host cells.

### 2.2. HDAC6^−/−^Mice are Transiently More Susceptible to Colonization by UPEC

**Figure 2:**
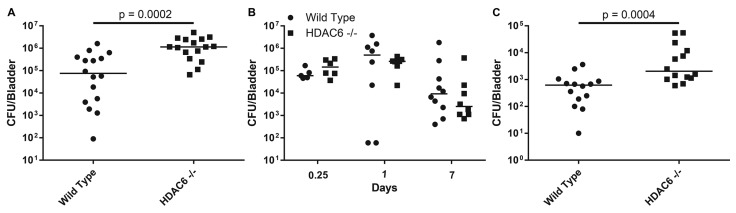
HDAC6 affects initial colonization of the bladder by UPEC, but not the levels of persistent bacteria. Adult female WT and HDAC6^−/−^ mice were inoculated with ~10^7^ CFU of UTI89 via catheterization. (**A**) Graphs show gentamicin-protected bacterial titers present in the bladders after a 90-min infection (**B**) Bacterial titers recovered at 6 h, 1 day, and 7 days post-catheterization are shown. (**C**) Graph indicates numbers of gentamicin-protected bacteria recovered after a 90-min infection that included three rinses with PBS at 20 min post-inoculat-ion. Bars indicate median values for each group; *n* ≥ 6 mice. *p* values were calculated using Mann-Whitney U tests.

To determine if HDAC6 affects UPEC colonization of the bladder *in vivo*, we employed a well-established murine UTI model system in which we infected adult female WT C57Bl/6 and HDAC6^−/−^ mice with UPEC via transurethral catheterization. Ninety minutes after inoculation, bladders were collected and treated with the host membrane impermeable antibiotic gentamicin in order to kill any extracellular bacteria. Surprisingly, bladders from HDAC6^−/−^ mice contained significantly higher numbers of intracellular bacteria than those from control WT animals (Figure 2A), in sharp contrast to results obtained in the cell culture-based assays (Figure 1B and [11]). Despite differences in intracellular titers early on, total numbers of bacteria recovered from the WT and mutant mice at later time points were similar (Figure 2B).

While extracting bladders during the course of these studies, we observed that HDAC6^−/−^ mice often appeared to retain more urine than their WT counterparts. This raised the possibility that longer retention of the inoculum by HDAC6^−/−^ mice could give UPEC a greater opportunity to invade the urothelium, resulting in higher titers of intracellular bacteria than seen with the WT animals. To test this hypothesis, WT and HDAC6^−/−^ mice were inoculated with UTI89 as usual, followed 20 min later with three rinses of each bladder with warm phosphate buffered saline (PBS) delivered and removed via catheterization. Bladders were collected 90 min after initiation of infection and treated with gentamicin to determine the levels of intracellular bacteria. Overall titers of intracellular bacteria recovered were markedly lower in both WT and HDAC6^−/−^ bladders following the rinses with PBS, and median differences between the two mouse genotypes became somewhat less distinct (compare Figure 2A,C). Specifically, in the un-rinsed bladders there is more than a 10-fold difference in the numbers of intracellular bacteria detected in the WT and HDAC6^−/−^ mice, whereas in the PBS-rinsed bladders the median difference is only about 2-fold. These observations indicate that greater retention of the initial inoculum is in part responsible for the higher numbers of intracellular bacteria recovered from the HDAC6^−/−^ mice at the 90 min time point.

### 2.3. HDAC6^−/−^ Mice Have Irregular Bladder Morphology and Larger Bladder Volume Capacity

**Figure 3:**
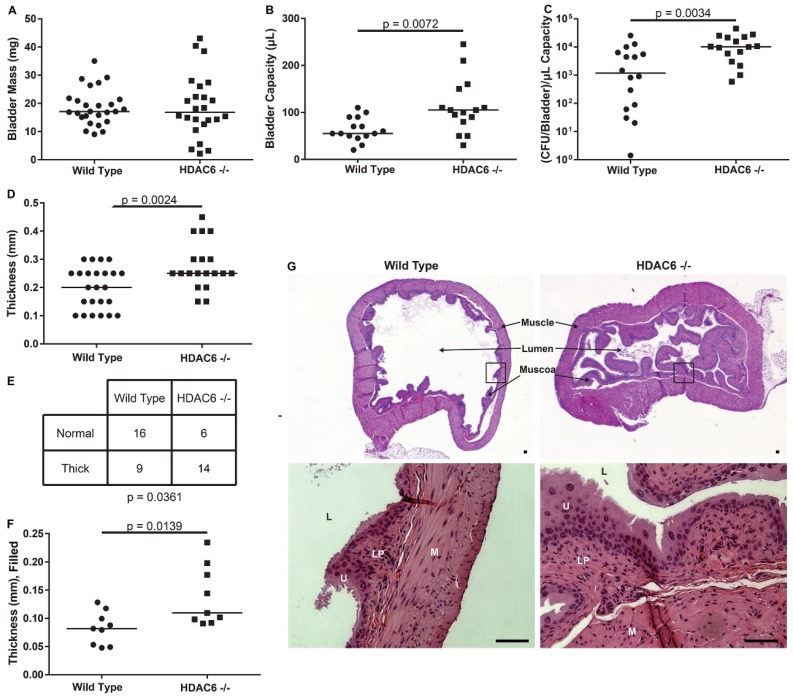
HDAC6 deletion alters bladder architecture and volume capacity. (**A**) Plot shows masses of bladders recovered from WT and HDAC6^−/−^ mice; (**B**) Graph depicts volume capacity measurements of bladders from uninfected WT and HDAC6^−/−^ mice; (**C**) Data from Figure 2A normalized to bladder capacity; (**D**) Plot shows results from muscle layer thickness measurements of bladders that were not filled at the time of fixation; (**E**) Contingency table in which muscle layer thickness observations are binned relative to mouse genotype; *p* value calculated using a two-tailed Fischer’s exact test; *n* = 25 bladder sections from 4 to 5 WT and mutant mice; (**F**) Plot shows results from muscle layer thickness measurements from bladders that were filled at the time of fixation. Measurements were taken at 3 points for each section and averaged; n ≥ 3 mice (with at least 3 sections per bladder); (**G**) Representative H&E-stained bladder sections. Scale bars = 50 µm. M, muscle; U, umbrella cells; L, lumen; LP, lamina propria. For graphs in A-D and F, bars indicate median values. *p* values indicated in these graphs were calculated using unpaired Student’s *t*-tests with Welch’s correction, as appropriate.

Bladders taken from age-matched WT and HDAC6^−/−^ female mice have similar masses (Figure 3A), but those from the mutant animals can accommodate substantially larger volumes of PBS introduced via catheterization (Figure 3B). Interestingly, even when intracellular UPEC titers (as shown in Figure 2A) are normalized to mean bladder volumes, the HDAC6^−/−^ bladders still contain significantly greater numbers of intracellular bacteria at the 90-min time point (Figure 3C). Likewise, normalization of the other data presented in Figure 2 to mean bladder titers did not alter the significance of the observed trends and did not affect our conclusions (not shown).

**Figure 4:**
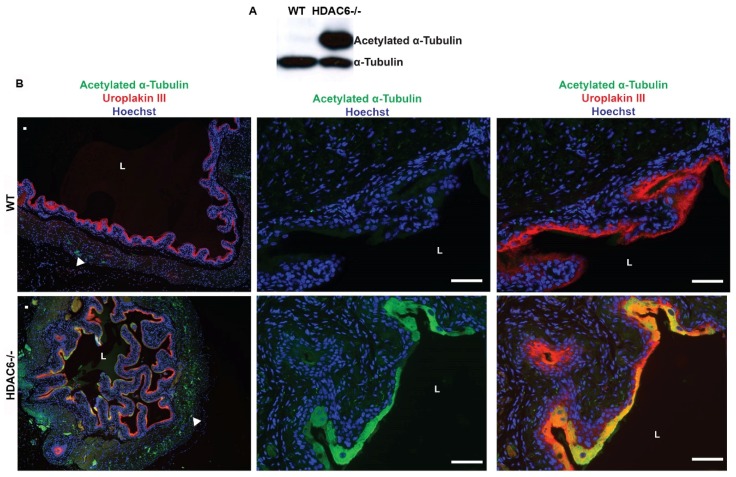
HDAC6 deletion results in increased levels of acetylated tubulin within the superficial umbrella cell and smooth muscle layers of the bladder. (**A**) Western blots showing levels of α-tubulin and acetylated α-tubulin in the mucosal layers of WT and HDAC6^−/−^ bladders; (**B**) Fluorescent micrographs of bladder sections from WT and HDAC6^−/−^ mice stained as indicated to visualize acetylated α-tubulin (green), uroplakin III (red), and nuclei (blue). Arrowheads highlight examples of acetylated α-tubulin within the smooth muscle layers. L, lumen. Scale bars = 100 µm.

Blinded analysis of hematoxylin and eosin (H&E)-stained bladder sections by a pathologist revealed that the smooth muscle layers of the bladders are, in general, significantly thicker in HDAC6^−/−^ mice than in WT animals (Figure 3D). Further evaluation of the histological data using a 2 by 2 contingency table confirmed that the correlation between the thickness of the bladder smooth muscle layer and mouse genotype is significant (p = 0.0361; Figure 3E). Measurements made using bladders that were filled at the time of fixation verified that the outer walls of bladders from HDAC6^−/−^ mice are often notably thicker than those from WT animals (Figure 3F). In addition, we found that the urothelium in HDAC6^−/−^ mice frequently displays a prominent papillary configuration demarking a more irregular and serrated bladder lumen than seen in WT animals (Figure 3G, see also Figure 4B). These alterations may reflect enlarged mucosal surface areas in the HDAC6^−/−^ mice, which could provide more sites for UPEC entry than are available in WT bladders.

Western blot analysis showed that the bladder mucosa in both HDAC6^−/−^ and WT mice had similar amounts of α-tubulin, whereas levels of acetylated α-tubulin where substantially elevated in mucosal tissues from mutant mice (Figure 4A). Immunofluorescence microscopy confirmed this finding, revealing that the HDAC6^−/−^ mice have especially high levels of acetylated α-tubulin within the superficial umbrella cells that line the lumen of the bladder (Figure 4B). These terminally differentiated epithelial cells, which are embedded with uroplakin complexes, are primary targets of UPEC following entry into the urinary tract [5]. The smooth muscle layers of bladders from the HDAC6^−/−^ mice also had increased amounts of acetylated α-tubulin, distributed throughout the tissue with scattered foci of more intense staining (see low magnification images in Figure 4A). Cumulatively, these results indicate that deletion of HDAC6 leads to alterations in both the morphology and volume capacity of the bladder, effects that are possibly due to aberrant increases in the levels of acetylated α-tubulin and perhaps other HDAC6 substrates. 

### 2.4. Neutrophil Recruitment Is Similar, But HDAC6^−/−^ Neutrophils Have More Bacteria

**Figure 5:**
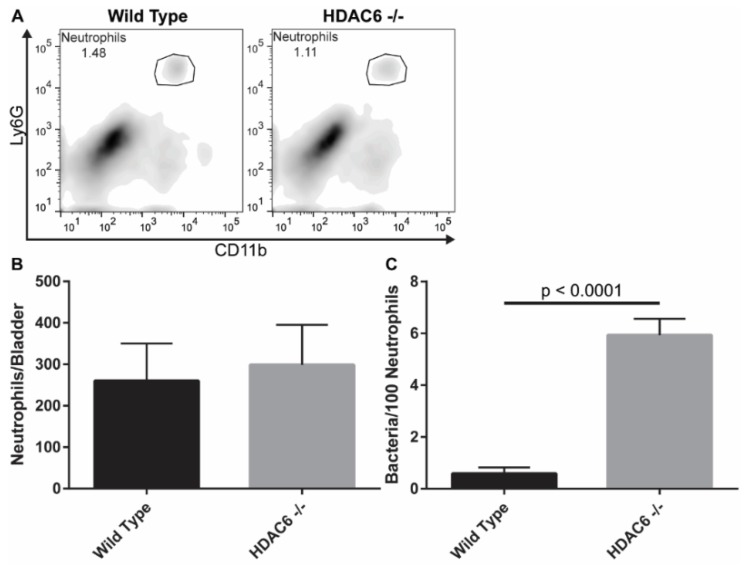
Neutrophils recovered from the bladders of infected HDAC6^−/−^ contain higher numbers of viable bacteria. (**A**) Representative flow plots of bladder neutrophils recovered from the bladders of WT and HDAC6^−/−^ following a 90-min infection with UPEC. Neutrophils were identified by gating on CD45^+^ populations and then on CD11b^+^Ly6G^+^ cells; (**B**) Numbers of neutrophils sorted from WT and HDAC6^−/−^ bladders; (**C**) Numbers of viable UPEC recovered from neutrophils after sorting from WT and HDAC6^−/−^ bladders. Data in (**B**) and (**C**) are expressed as the means ± SEM of 5 independent experiments. *p* values were determined using unpaired Student’s *t* tests.

One of the earliest immune cells recruited into the bladder in response to infection are neutrophils, which have been shown to be the primary phagocytes responsible for bacterial clearance during acute UTI [37,38]. In considering the importance of neutrophils, we set out to determine if the heightened levels of intracellular UPEC that we detected early on in the HDAC6^−/−^ mice might also be partly attributable to defective neutrophil responses. At 90 min post-inoculation with UTI89∆*hlyA*, bladders from WT and HDAC6^−/−^ were collected and the neutrophils present were stained and sorted using antibodies specific for the surface markers CD45, CD11b, and Ly6G (Figure 5A). WT and HDAC6^−/−^ bladders contained similar numbers of neutrophils at the 90-min time point (Figure 5B). However, viable bacterial counts were significantly higher in neutrophils recovered from the bladders of HDAC6^−/−^ mice (Figure 5C). These results indicate that the infiltrating neutrophils from HDAC6^−/−^ mice either (1) kill UPEC more slowly or (2) are simply exposed to more bacteria within the bladder and therefore have the opportunity to phagocytose a greater number of pathogens. Interestingly, in a mouse model of septic shock, use of the HDAC6 inhibitor tubastatin A can reduce myeloperoxidase (MPO) production by peritoneal neutrophils, supporting the idea that HDAC6 can affect neutrophil-associated bactericidal activities [24].

## 3. Experimental Section

### 3.1. Chemicals, Reagents, and Antibodies

Dulbecco’s Modified Eagle Medium (DMEM) High Glucose, Gentamicin, heat inactivated fetal bovine serum (∆FBS), mouse anti-acetylated α-tubulin (clone: 6-11B-1), neutral buffered formalin, Triton X-100, and PBS were obtained from Sigma-Aldrich. Lysogeny Broth (LB) and ethylenediamine tetraacetic acid (EDTA) were purchased from Fisher Scientific. Anti-mouse CD45 conjugated to BV421 (clone: 30-F11), anti-mouse CD11b conjugated to Alexa Fluor 488 (clone: M1/70), and anti-mouse Ly6G conjugated to APC (clone: 1A8) antibodies were purchased from BioLegend. FcBlock (clone 2.4G2) was obtained from BD Pharmigen. Mouse anti−α-tubulin (clone B-5-1-2) and donkey anti-mouse Alexa Fluor 488 were purchased from Abcam. Donkey anti-rabbit Alexa Fluor 555 and Collagenase IV were purchased from Life Technologies, while DNase I and Protease Inhibitor Cocktail are from Roche. Histo-Clear was acquired from National Diagnostics, and Bovine serum albumin (BSA) was bought from Gold Biotechnology. Rabbit anti-uroplakin III was kindly provided by Henry Sun (New York University). 

### 3.2. Bacterial and Cell Culture

The human cystitis UPEC isolate UTI89 or UTI89∆*hlyA* has been described previously [14]. This strain colonizes the mouse urinary tract similar to WT UTI89, but induces less tissue damage. Bacteria were grown statically from frozen stock in 20 mL LB at a 37 °C in an air incubator for 24 h (for the *in vitro* studies) or 48 h (for use in the mouse experiments). WT and HDAC6^−/−^ MEFs were grown in DMEM + 10% ΔFBS at 37 °C in a humidified incubator with 5% CO_2_.

### 3.3. Mouse Strains and Genotyping

WT C57BL/6J mice were purchased from The Jackson Laboratory. HDAC6^−/−^ mice generated on the C57BL/6J background were kindly provided by Patrick Matthias at the Friedrich Miescher Institute [39]. All mice used in experiments were 8–10 week old females. The HDAC6^−/−^ genotype was confirmed using the following primers, as described [39]: HDAC6-F, 5’-GTACAATGTGGCTCACAGAA-3’; HDAC6-WT-R, CAGGCACAGGA ATATGAGTT; HDAC6-KO-R CAACTCTGCCTCTCCTGGAT. Animals were used according to protocols approved by the Animal Studies Committee at the University of Utah.

### 3.4. Gentamicin Protection Assays

WT and HDAC6^−/−^ MEFs were plated into 24-well tissue culture plates and grown overnight to confluency. Triplicate wells were infected with UTI89∆*hlyA* at a multiplicity of infection of ~15 CFU. Plates were centrifuged at 300× *g* to synchronize bacterial contact with the host cells, and subsequently incubated for 60 min at 37 °C in a humidified incubator with 5% CO_2_. Wells were then washed with DMEM + 10% ΔFBS. The numbers of host cell-associated bacteria (adherent and invaded microbes) were determined at this point by lysing the host cells in 1 mL PBS + 0.3% Triton X-100 and plating serial dilutions of the lysates on LB agar. Other monolayers, after washes, were incubated with DMEM + 10% ΔFBS + 100 µg/mL gentamicin for an additional 60-min incubation, followed by washes with DMEM + 10% ΔFBS and lysis in PBS + 0.3% Triton X-100. Surviving bacteria, recovered from intracellular populations, were enumerated by plating serial dilutions of the lysates. Data were normalized by dividing the number of intracellular bacteria by the total number of cell-associated bacteria to account for any differences in host cell numbers. Levels of intracellular (% Invasion) are expressed relative to control WT samples. Assays were repeated in quadruplicate.

### 3.5. Bladder Catheterization

Mice were anesthetized by isofluorane inhalation and catheterized with a 50-µL suspension of UTI89 (~10^7^ CFU) in PBS using a lubricated catheter attached to a 1 cc syringe via a 30 G needle. After catheterization, mice were kept under anesthesia on their side for 10 min, and rotated to their other side for an additional 10 min before being returned to their cages. In some experiments, bladders were rinsed with PBS prior to returning the mice to their cages. At the indicated time points, animals were sacrificed under anesthesia by cervical dislocation and bladders were removed aseptically and quartered prior to homogenization. For the *ex vivo* gentamicin protection assays, bladders collected and quartered after the 90-min time point were incubated for 60 min at 37 °C in PBS + 100 µg/mL gentamicin, washed 3X with PBS, and homogenized using a BulletBlender Storm 24 tissue homogenizer (Next Advance) with 3.2 mm stainless steel balls. Serial dilutions of the homogenates were plated on LB agar to determine bacterial counts per bladder. Cumulative data from two or more independent assays are presented. 

### 3.6. Bladder Capacity Measurements

Adult female WT and HDAC6^−/−^mice were anesthetized with isofluorane and catheterized with warmed PBS to fill the bladder until backflow was observed. A second catheter was then carefully inserted to extract and measure the volume of PBS remaining within each bladder. This process was repeated on 3 different days.

### 3.7. H&E Staining and Immunofluorescence

Bladders from uninfected mice were extracted and fixed in 10% neutral buffered formalin for 24 h. Alternatively, prior to extraction from mice, bladders were filled via catheterization with 50 μL 10% neutral buffered formalin and allowed to fix for 20 min before being removed and immersed in formalin. All bladders were sent to the University of Utah Research Histology core for paraffin embedding and sectioning at random levels. The core performed H&E staining of sections. For immunofluorescence, slides were deparaffinized by three washes with Histo-Clear following by rehydration in a series of incubations with decreasing concentrations of ethanol (100%, 70%, 50%, 45%, and 30%). Blocking and permeabilization steps were performed using PBS + 3% BSA + 0.5% Triton X-100, followed by primary staining with anti-acetylated α-tubulin and anti-uroplakin III. Secondary staining was performed with Alexa Fluor 488- or 555-conjugated anti-mouse and anti-rabbit antibodies. Sections were then stained using Hoechst dye (1 mg/mL), and following final washes in PBS the slides were mounted using FluorSave Reagent. Fluorescent and H&E-stained sections were imaged using an Olympus BX51 microscope equipped with a QImaging QIClick cooled CCD camera. Bladder wall thickness measurements were acquired by averaging 3 measurements taken across each bladder section by blinded investigators.

### 3.8. Western Blotting

Bladders from WT and HDAC6^−/−^ mice were extracted, halved longitudinally, splayed, and pinned down lumenal side up in PBS on silicon discs (Sylgard 184 silicone elastomer, Dow Corning Corp.). The mucosa was then carefully removed using fine point tweezers, and proteins were extracted using a dounce homogenizer and RIPA buffer containing a protease inhibitor cocktail. 50 µg of protein from each sample was resolved in 12.5% polyacrylamide gels by Tris-Glycine SDS-PAGE. Proteins were then transferred to an Immobilon PVDF-FL membrane (Millipore) and blocked with Tris-Buffered Saline (TBS) + 5% Non-Fat Milk Powder. Blots were incubated overnight at 4 °C with primary antibodies (anti-α-tubulin or anti-acetylated α-tubulin), washed, and subsequently probed and visualized using horseradish peroxidase-conjugated secondary antibodies and the SuperSignal West Femto substrate (ThermoFisher Scientific).

### 3.9. Bladder Dissociation and Flow Cytometry

WT and HDAC6^−/−^ mice were catheterized with UTI89∆*hlyA* and 90 min later bladders were collected and dissociated into a single-cell suspensions as previously described [13]. Briefly, bladders were minced using surgical scissors and incubated for 40 min in RPMI1640 + 0.5% ∆FBS + 20 m*M* HEPES + 1 mg/mL Collagenase IV + 0.1 mg/mL DNase I. Suspensions were strained through a 70-µm nylon mesh and washed 3X with flow buffer (PBS + 0.5% BSA + 25 m*M* HEPES + 1 m*M* EDTA). Suspensions of equal numbers of cells were blocked for 15 min with FcBlock (BD Biosciences) and then stained with antibodies against CD45, CD11b, and Ly6G for 30 min. Samples were analyzed on a FACSAria II and CD45^+^CD11b^+^Ly6G^+^ cells were sorted into PBS + 0.3% Triton X-100. Serial dilutions were plated on LB agar to enumerate numbers of neutrophil-associated bacteria.

### 3.10. Statistics

Prism 6.07 (GraphPad Software, Inc.) was used for all statistical tests. *p* values of less than 0.05 were defined as significant.

## 4. Conclusions

Results presented here indicate that HDAC6^−/−^ mice are able to deal with UTIs much like their WT counterparts in the hours and days following initiation of infection. However, the mutant mice do contain markedly higher levels of intracellular bacteria at an early time point after inoculation of the bladder with UPEC. These results contrast with those obtained in cell culture-based assays in which host cell invasion by UPEC was inhibited when HDAC6 was deleted, silenced, or pharmacologically suppressed (see Figure 1 and [11]). This discrepancy may be in part attributable to the development of compensatory pathways in mice that lack HDAC6. In support of this argument, we note that HDAC6^−/−^mice develop fairly normally despite having systemically high levels of acetylated α-tubulin and Hsp90, and regardless of the sizable detrimental effects that HDAC6 interference has on diverse cellular functions *in vitro* [12,15,39]. Though not tested here, it is also possible that HDAC6 deletion increases host receptor availability and/or modifies the intracellular trafficking and autophagic pathways that control the internalization and efflux of UPEC by bladder epithelial cells *in vivo* [5,40,41]. In addition, the differentiation status of the host cells that are targeted by UPEC may influence the net effect of HDAC6 deletion on UPEC entry. In HDAC6^−/−^ mice, the terminally differentiated umbrella cells have especially high levels of acetylated α-tubulin relative to other cells within the bladder mucosa (see Figure 3G). Whether or not this change specifically affects the internalization of UPEC by the umbrella cells is not yet known. Despite these ambiguities, we did uncover unanticipated roles for HDAC6 as a regulator of bladder architecture and function. Specifically, our data indicate that deletion of HDAC6 promotes the elaboration of papillary structures by the bladder mucosa and increases the thickness of the smooth muscle layers. These effects likely contribute to the greater volume capacity and fluid retention that we observed with bladders from the HDAC6^−/−^ mice, and may provide UPEC with more opportunities to bind and invade the bladder mucosa. This in turn may bring increased numbers of UPEC into contact with infiltrating neutrophils that may have reduced anti-bacterial activities. Slower killing by neutrophils may help explain why bacterial titers within the HDAC6^−/−^ bladders eventually come down to levels seen in the WT mice. Cumulative changes in neutrophil activities as well as bladder architecture and function may counter any inhibitory effects that the lack of HDAC6 has on UPEC entry into individual bladder epithelial cells. These results highlight the complexities of HDAC6 as a regulator of both bladder physiology and bacterial pathogenesis, and raise the possibility that HDAC6 dysregulation may be involved in other urological disorders, such as urinary retention. Discerning the specific effects of HDAC6 as a regulator of urinary function and host cell invasion by UPEC will likely require additional tools, such as inducible, urothelial-specific HDAC6 knockout mice.
